# Editorial: How the Timing, Nature, and Duration of Relationally Positive Experiences Influence Outcomes in Children With Adverse Childhood Experiences

**DOI:** 10.3389/fnbeh.2021.755959

**Published:** 2021-09-06

**Authors:** Erin P. Hambrick, Soraya Seedat, Bruce D. Perry

**Affiliations:** ^1^Department of Psychology, University of Missouri – Kansas City, Kansas City, MO, United States; ^2^Department of Psychiatry, Faculty of Medicine and Health Sciences, Stellenbosch University, Cape Town, South Africa; ^3^Department of Psychiatry and Behavioral Sciences, Feinberg School of Medicine, Northwestern University, Chicago, IL, United States; ^4^School of Allied Health College of Science, Health and Engineering, La Trobe University, Melbourne, VIC, Australia

**Keywords:** relational health, resilience, child trauma, adverse child experiences, positive childhood experiences

The role of quality relationships in children's lives, particularly children who have experienced trauma or adversity, has recently become more fully appreciated. Studies on the negative effects of child trauma and adversity, and their health impacts across the life-course, have proliferated in recent decades and led to greater societal awareness and public-policy efforts to both prevent trauma and adversity and decrease its long-term effects. Focusing on resilience-promoting child protective factors, more specifically the quality of children's relationships in a variety of contexts or improving their “relational health,” may be just as effective at not only preventing trauma but buffering its effects. Yet this line of investigation has received relatively scant attention. Indeed, developing and nurturing positive relationships and reducing barriers to relational health in early life may even be a more feasible (and reaffirming) approach. A recent revised policy statement on childhood toxic stress issued by the American Academy of Pediatrics (Garner et al., 2021, pp. 1) endorsed “a paradigm shift toward relational health because safe, stable and nurturing relationships not only buffer childhood adversity when it occurs but also promote the capacities needed to be resilient in the future.” This special topic highlights recent advances in the study of the role of relationally healthy experiences in promoting health and resilience even when adverse or traumatic childhood experiences are part of the picture.

## Influence of Relational Health

The field of infant mental health has raised awareness of the importance of early parent-child relationships and the need to focus on building strengths in families instead of solely focusing on decreasing individual-level pathology (Berg and Lachman). In general, early positive relational experiences are associated with progress toward meeting developmental milestones, developing core regulatory processes and building foundational skills to adapt to future adversities. Lachman et al. coded video-recorded mother-infant interactions for shared pleasure, and shared pleasure was associated with less concurrent infant withdrawal and with better motor and cognitive development over time. Despite the potential importance of shared pleasure, its occurrence in the sample was low. A study by Rollins and Crandall examined the role of positive childhood experiences (PCEs), including parent-child connection and having a good friend, in influencing self-regulation, anxiety, depression, and substance use during adolescence and found that PCEs contributed to better self-regulation and less shame, which in turn was associated with fewer mental health problems—even when adversity was present.

## Specificity of Relational Experiences

While an overall recognition that relationships matter is important—knowing the type and nature of the most powerful relational experiences—and when during a person's development these experiences are most powerful—is key for promoting positive outcomes. Santini et al. found that multiple leisure activities per week during adolescence, including exercise, was associated with better well-being and less substance use—reinforcing the idea that consistency and repetition in positive experiences is key. In another study, Santini et al. found that during adolescence, social disconnectedness at school, such as lack of classmate and teacher support, is associated with decreased well-being and mental health problems—with the presence of just one type of school disconnection putting adolescents at risk. Moreover, while frequent, quality connection is probably always good—we must also root out potential sources of disconnection and address any context-specific barriers.

In assessing the link between childhood emotional neglect, or the absence of “parental attention and support,” and depression during adolescence, Glickman et al. noted that neglect was indeed associated with depression during adolescence, but this association was moderated by the presence of positive peer relationships. Additionally, the quality of relational experiences—particularly when both parties (e.g., parents and children) agree that the relationship is strong—can promote children's self-esteem and adjustment, even amongst high risk groups, such as the drug court participants studied by Guastaferro et al.

## Promoting Relational Health

This body of work conveys some key messages about what we can do now to improve children's relational health. Starting in the earliest days of a child's life is certainly key (Lachman et al., Berg and Lachman), as is the frequency and consistency of quality experiences. Ensuring positive experiences are available across domains (e.g., family, peers, school, community) and that the relational experiences are developmentally congruent, such as a focus on peers during the adolescent years and parents during the early years, may also show promise (Santini et al.; Glickman et al., Lachman et al.). Although relational health is a potentially potent prevention tool and should be embedded within a public health approach, it should also be an important emphasis of mental health services, particularly primary care level services for trauma-exposed youth (Cox et al.). In a sample of maltreated treatment-seeking youth, Cox et al. found that treatment services led to both improved relational and mental health, with, perhaps, relational health improvement promoting other observed clinical gains. Improving relational health may be a feasible, strengths-focused approach to improving well-being amongst all youth (Glickman et al.)—and it perhaps may most benefit those with trauma histories.

## Future Research, Clinical and Policy Directions

While it is promising and even exciting that relationships can have a potent and enduring influence, we cannot oversimplify this important issue. Just like the timing and nature of developmental trauma experiences uniquely influence children's developmental trajectories—the timing and nature of relational experiences will do the same. Some children, for example, with developmental trauma histories are “sensitized” to relational interactions—and the manner in which relationships are introduced therapeutically must be thoughtful and individualized. And, we cannot just say “provide children with better relationships” without developing systems that prioritize relationships during the prenatal period, infancy, and throughout childhood and adolescence. Who are the children, for example, who experience relational poverty, and what systemic issues are the root cause? And so, now that there is promise, there must be discipline in researching when and how relationships can be most preventive and healing, as well as toward ensuring equitable access to healthy relationships throughout development.

## Author Contributions

EH and SS drafted the editorial. BP served as the editor. All authors contributed to the article and approved the submitted version.

## Conflict of Interest

The authors declare that the research was conducted in the absence of any commercial or financial relationships that could be construed as a potential conflict of interest.

## Publisher's Note

All claims expressed in this article are solely those of the authors and do not necessarily represent those of their affiliated organizations, or those of the publisher, the editors and the reviewers. Any product that may be evaluated in this article, or claim that may be made by its manufacturer, is not guaranteed or endorsed by the publisher.
